# Multidisciplinary assessment of patients with ischemic stroke, the structure of a stroke team, and first Egyptian experience in adults undergoing transcatheter PFO closure for PFO-related stroke

**DOI:** 10.1186/s43044-021-00139-8

**Published:** 2021-03-06

**Authors:** Amr Mansour, Noha M. Gamal, Azza A. Elfiky, Mohamed Ayman Saleh, Samia Ashour Mohamed, Hala Mahmoud ElKhawas, Ahmed ElSadek, Noha L. Dawood, Maiy H. Elsayed

**Affiliations:** 1 Cardiology Department, Ain Shams University, Cairo, Egypt; 2 Cardiology Department, Faculty of Medicine, Assuit University Hospital, Assiut University, Assuit, 71515 Egypt; 3 Neurology Department, Ain Shams University, Cairo, Egypt

**Keywords:** Cryptogenic stroke, Transcatheter PFO closure, PFO-related stroke, Atrial septal aneurysm, Fenestrated interatrial septum

## Abstract

**Background:**

Patent foramen ovale closure in the setting of stroke was debatable until the recent data from the long-term follow-up of multiple randomized control trials. These recent data have led to increase the number of the procedure worldwide.

To our knowledge, there was no previous formal structured program in Egypt between cardiologists and neurologists for investigation and management of patients with cryptogenic stroke.

The first Egyptian-dedicated stroke team was created in two large tertiary centers with collaboration between cardiologists, dedicated cardiac imagers, and neurologists for investigation and management of patients with cryptogenic stroke.

**Results:**

Sixty-three patients with cryptogenic stroke were identified from a total of 520 patients admitted to the stroke units between 2016 and 2019. Twenty-five patients had a proven PFO-related stroke. Three patients were referred for surgical closure, 19 patients underwent transcatheter PFO closure, and procedural success was met in 18 patients (94.7%). We did not experience any major procedure-related complication. Complete closure was achieved in 83.3% of patients at 6 months. One patient had a single attack TIA within the first 3 months after device closure; one patient had a device-related thrombosis; both were managed successfully.

**Conclusion:**

Our initial experience in collaboration between cardiologist and neurologist with the establishment of a dedicated cryptogenic stroke team added significantly to the management of patients with stroke.

The results of the first Egyptian cohort who underwent transcatheter PFO closure demonstrated procedural feasibility, safety, and efficacy with very low incidence of major complications.

A nationwide program is needed to reduce the ischemic stroke disease burden and the risk of recurrence.

**Supplementary Information:**

The online version contains supplementary material available at 10.1186/s43044-021-00139-8.

## Background

Stroke is the second cause of death and disability worldwide [1] with almost one third of the ischemic strokes is of unknown etiology, i.e., cryptogenic [2].

Patent foramen ovale (PFO) has high incidence in patients with cryptogenic stroke reaching up to 40% in some registries [2]. This has generated long investigations and debate on its propensity for stroke by paradoxical embolism and its management for stroke prevention.

PFO results from incomplete fusion between the septum primum and septum secundum leading persistent communication between the right and left atrium forming a flap valve that opens when the right atrial pressure exceeds the left atrial pressure by maneuvers that change the intrathoracic pressure (e.g., sneezing, coughing, or straining), allowing the PFO to open, and blood, thrombus, or any other substance to pass across from the right to left atrium causing a paradoxical embolus. This transfer is associated with several clinical phenomena, including cryptogenic stroke [3]. There are various anatomical variations in the morphology of PFO, according to the PFO tunnel length, thickness of septum secundum, presence or absence of other fenestration, and atrial septal aneurysm, with large variation in its size [4].

Cumulative evidence from recent multiple randomized control trials has led to the FDA approval of transcatheter PFO closure in patients less than 60 years for prevention of recurrent stroke after careful patient assessment, and this resulted in increase of the number of the performed procedures worldwide [5].

PFO closure in adults for prevention of recurrent strokes was rare and in unique individual circumstances in Egypt; this was due to the combination of the decreased awareness of the recent trial data among both the referring neurologists and cardiologists and the lack of reimbursement of the procedure by the Egyptian medical system with no institutional structured dedicated collaboration between these subspecialities.

We thought to describe the first Egyptian stroke team experience in the evaluation and management of patients with cryptogenic stroke and the results of the first Egyptian cohort who underwent transcatheter PFO closure.

## Methods

Ethical approval was obtained from our institutional local ethical committee, and informed written consent was obtained from all the participants. The study was conducted in two large tertiary centers in Egypt.

We established a conjoint, structured program for evaluation, investigation, and management of patients with stroke between the neurology and cardiology departments in the study centers with dedicated cryptogenic stroke teams.

The members of the stroke team included the neurgologist, stroke specalists, dedicated cardiac imager, cardiologists, and interventional cardiologists. Other specialities such as hematologist, cardiothoracic surgeon, and rheumatologists were involved when indicated.

**Fig. 1:**
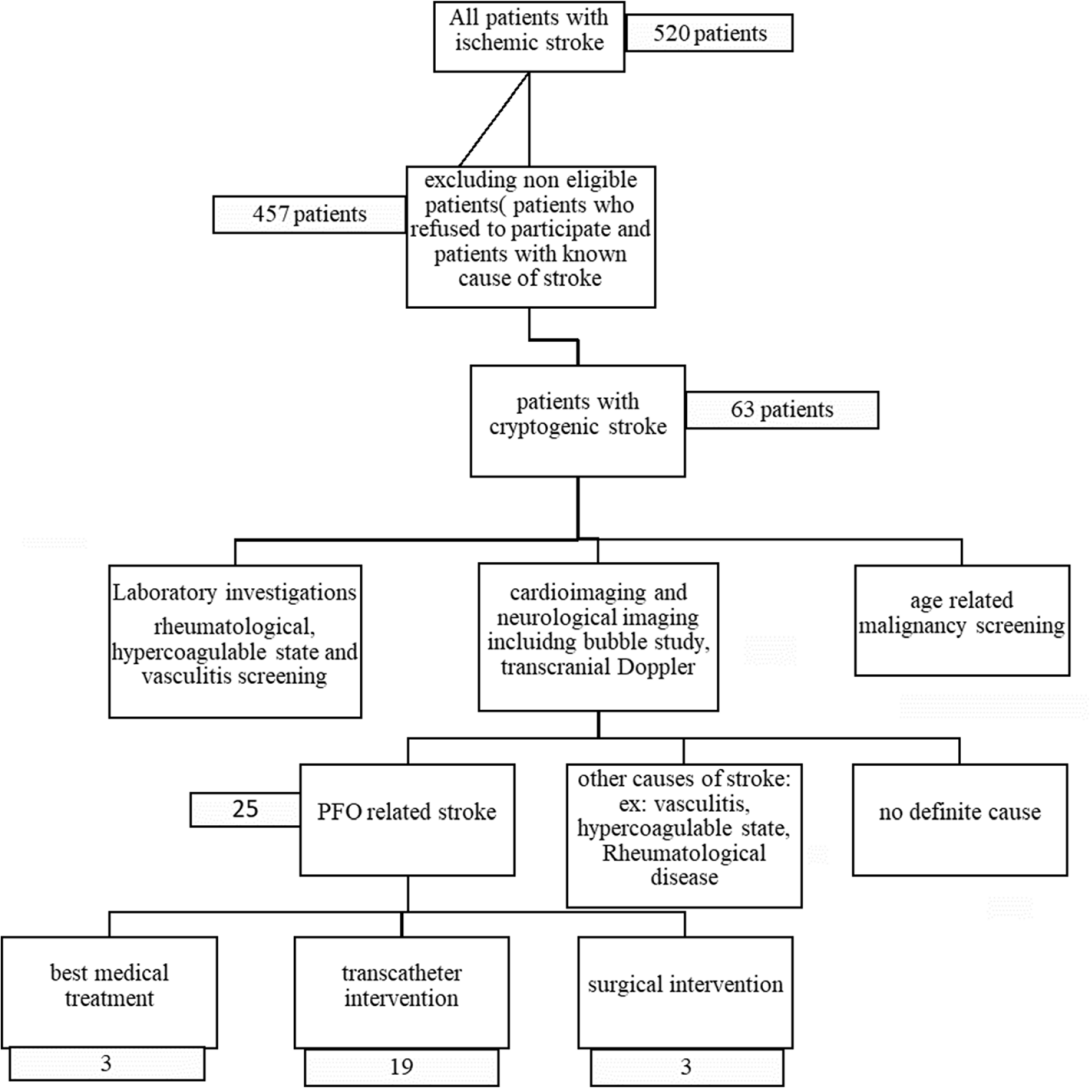
Patient enrollment and study flow chart

All patients with ischemic stroke admitted between 2016 and 2019 were enrolled in the study (Fig. 1).

We excluded patients with hemorrhagic stroke, identifiable cause of stroke, dropped out patients during the investigation process, and patients who refused to participate in the study. Eligible patients were subjected to the following:

- ▪ Initial evaluation by:
- ▪ Thorough history taking with special emphasis on cardiovascular risk factors, vital sign assessment, and thorough general and neurological examination including assessment of severity of neurological deficit using NIH stroke scale (NIHSS) [6] and modified Rankin scale (mRs) for handicap [7].
- ▪ Immediate investigation including 12 leads electrocardiogram (ECG) and non-contrast CT brain.
- ▪ Systemic arterial hypertension was excluded by revision of the patient’s previous blood pressure recordings if present, serial in-hospital and out of office blood pressure measurement recordings. Ambulatory blood pressure monitoring was done in suspected cases when clinically indicated.
- ▪ Neurological imaging including computed tomography angiography (CTA) head and neck, magnetic resonance angiography (MRA) head and neck, magnetic resonance imaging (MRI) brain, and carotid ultrasound.
- ▪ Laboratory investigations: basic laboratory investigations including complete blood count (CBC), liver function test, kidney function tests, electrolytes, erythrocyte sedimentation rate (ESR), C-reactive protein quantitative level (CRP), fasting blood sugar (FBS), glycated hemoglobin level (HBA1c), lipid profile, and oral glucose tolerance test.
- ▪ Basic cardiac imaging: transthoracic echocardiography (TTE).
- ▪ Patients who had negative tests results were classified to be of undetermined etiology according to TOAST classification [8]. Those patient had further investigations and workup including rheumatological panel and vasculitis screen, anti-nuclear antibodies (ANA), anti-deoxyribonucleic acid antibodies (anti-DNA), anticardiolipin antibody, lupus anticoagulant, and antineutrophil cytoplasmic antibodies (ANCA-P and ANCA C).
- ▪ Hypercoagulable state assessment: protein C, protein S, antithrombin III deficiency, prothrombin gene mutation, factor V Leiden, lupus anticoagulant, anticardiolipin antibodies, and homocysteine level.
- ▪ Age-appropriate malignancy screening was done on clinical suspicion and when indicated.
- ▪ Transthoracic and transesophageal echocardiography: a specific stroke protocol with bubble study to evaluate the interatrial septum (IAS) for the presence of atrial septal aneurysm (ASA) which was defined as ≥ 10-mm midline shift in anatomical M-mode or when total bidirectional shift was > 15 mm [9]. Patent foramen ovale (PFO); interatrial septal fenestrations; atrial septal defects; intracardiac tumors or masses; left atrial appendage thrombi; assessment of the cardiac valves, ascending aorta, aortic arch, and descending aorta for any plaques, or other pathologies.
- ▪ Detection of intracardiac shunts was done at rest and by using abdominal compression and/or Valsalva maneuver. The Valsalva maneuver begun when the contrast filled the right atrium and was maintained for > 5 s. Intracardiac shunt was defined as the appearance of bubbles in the left-sided cardiac chambers within the first 3 cardiac cycles after opacification of the right atrium, whether at baseline or after the Valsalva maneuver. The degree of shunt was quantified according to the numbers of bubbles into: absence of shunt, mild (<10 microbubbles), moderate (10–20 microbubbles), or severe (>20) [9].
- ▪ Cardiac rhythm monitoring: 72 h cardiac telemetry monitoring to exclude any paroxysmal arrhythmia.
- ▪ Contrast enhanced Transcranial Doppler (c-TCD): for detection of micro-embolic signals in middle cerebral artery for confirmation of presence of right to left shunting by using agitated saline injected into the antecubital vein via a three-way stopcock immediately after contrast preparation. If no microbubbles were detected after the first injection, then further two injections were made with a Valsalva maneuver. Micro embolic signals were considered positive if detected within 30 s from contrast injection and their severity were graded [6]. We applied a 4-level visual classification for c-TCD test: no occurrence of micro-embolic signals, grade I 1 to 10 signals, grade II 10 to 30 signals but not curtain, and grade III curtain pattern [10, 11].

Patients in whom PFO was proven to be incriminated in the pathogenesis of stroke with direct causation relationship and not just an association—by exclusion of other etiologies, and demonstration of right to left shunt across the PFO—were then subjected to a multi-disciplinary team discussion with active involvement of the patient himself to determine the best management strategy in accordance with the patient preference.

## Results

The study included a total of 520 patients with ischemic stroke who were admitted to the stroke units between 2016 and 2019. After excluding patients with identifiable causes of stroke and patients who were lost during the investigational procedures, a total number of 63 patients with cryptogenic stroke were eligible for further work up (Fig. 1).

Patients with cryptogenic cerebrovascular stroke included 36 males (57.1%) and 27 females (42.8%). Their age ranged from 20 to 59 years with mean age of 36.1 ± 9.3 years.

Twenty-four patients had a previous history of one or more stroke or transient ischemic attack (TIA) (38.09%).

Seven patients tested positive for lupus anticoagulants (11.1%), and 3 patients tested positive for anticardiolipin antibodies (4.7%). Eight patients had protein C deficiency (12.6%), 9 patients had protein S deficiency (14.2), 4 patients had antithrombin III deficiency (6.3%), one patient was diagnosed with Takayasu arteritis, and one patient had systemic lupus erythematosus (SLE). We had one patient with concomitant deep venous thrombosis, pulmonary embolism, and stroke.

### Site of cerebrovascular stroke on neuroimaging studies

Fifty-one ischemic strokes were located anterior, i.e., in the distribution of anterior cerebral artery or middle cerebral artery (80.9%), and 12 ischemic strokes were located posterior in the distribution of the vertebral, basilar, or posterior cerebral artery (19.1%).

### Cardiac imaging studies in patients with cryptogenic stroke

Four patients had intracardiac thrombi (6.3%), 5 patients had descending aorta plaques (7.9%) only one of them had complex aortic plaque (which is defined as increased echo density and thickening of the intima > 5 mm with shaggy overlying echogenic material and marked irregularities in the intimal wall), and 2 patients had filamentous mass in relation to aortic valve (Lamble’s excrescence) (3.1%).

### Assessment of the interatrial septum

Twenty-six patients had patent foramen ovale PFO (41.2%) by trans-esophageal echocardiography, 13 of them had associated atrial septal aneurysm (ASA), 5 had associated fenestration, and 1 patient had secundum atrial septal defect.

Twenty-four patients had positive shunting on bubble study on TTE and/or TEE (using agitated saline, Valsalva maneuver, and/or abdominal compression). The degree of shunting by TEE was mild in 5 patients, moderate in 13 patients, and severe in 6 patients.

#### Contrast transcranial Doppler

We had 5 patients with severe degree of shunt on TCD at rest, 16 patients with grades II and III shunts, and 4 patients with grade I shunt.

One patient with PFO showed evidence of right to left shunt on c-TCD only, and one patient did not reveal any evidence of right to left shunt on both TEE and c-TCD.

### Management

We included the 25 patients with PFO and evidence of right to left shunt in active team discussion to determine the best management plan.

### Surgical repair

Three female patients were referred for surgical repair of the interatrial septum due to multiple fenestrations occupying the whole septum; two of them had a very large, highly mobile ASA that was deemed not to be suitable for transcatheter closure (video 1).


**Additional file 1: Video 1.** 2 D trans-esophageal echocardiography in mid esophageal four chambers view showing patent foramen ovale (PFO) with very large highly mobile Atrial septal aneurysm (ASA).

### Medical management

Four patients refused transcatheter closure and opt to medical treatment. They were kept on dual antiplatelet therapy (DAPT) therapy including acetylsalicylic acid and clopidogrel. One patient had recurrence TIA within the first 2 months of medical treatment, and he was crossed over to the transcatheter closure arm.

### Transcatheter closure

Nineteen patients underwent transcatheter PFO closure. The procedure was done under general anesthesia and trans-esophageal echocardiography guidance in all patients.

Right femoral vein access was used in all patients. Intravenous dose of 100 IU/Kg heparin was given after securing the vascular access; activated clotting time (ACT) was monitored in all the patients with a target level more than 250 s; additional heparin doses were given if needed according to the ACT. All sheaths and catheters were routinely flushed with heparinized saline (1 IU/ml); manual compression was used in all patients after sheath removal.

We used balloon interrogation and sizing of the PFO tunnel in 15 patients (78.9%).

**Fig. 2:**
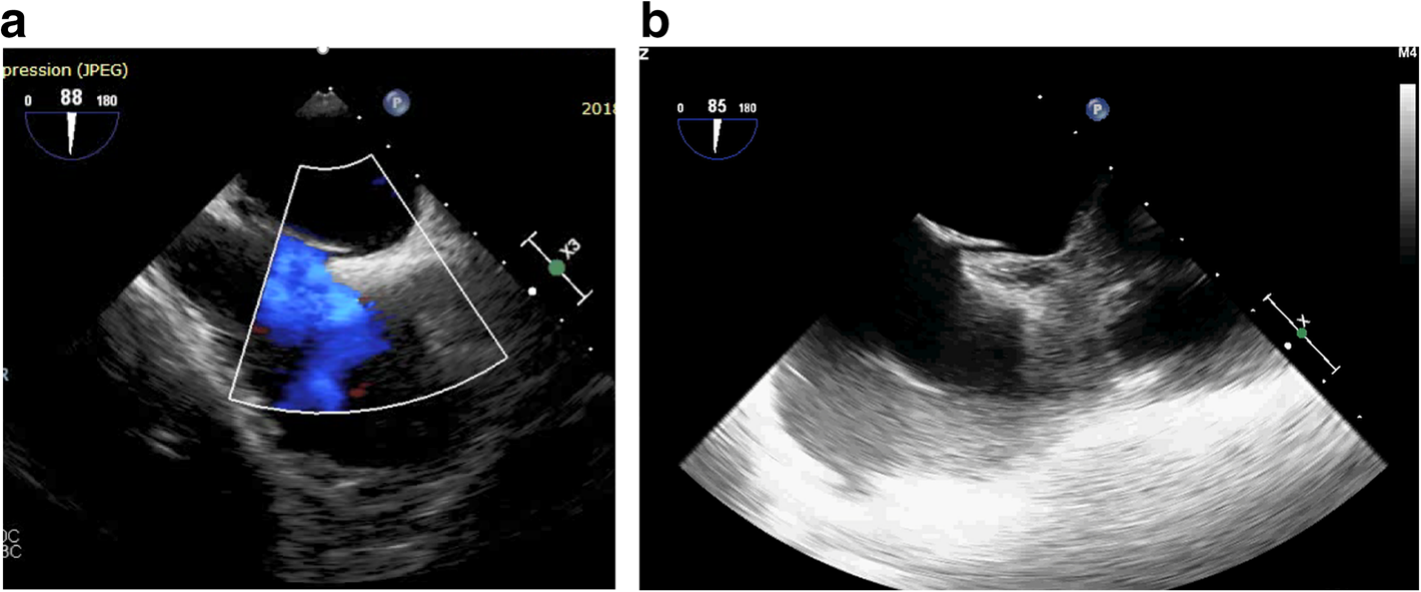
**a** 2D trans-esophageal echocardiography with color flow mode in bicaval view showing tunnel-like patent foramen ovale (PFO). **b** 2D trans-esophageal echocardiography without color flow mode in bicaval view showing tunnel-like patent foramen ovale (PFO)

**Fig. 3:**
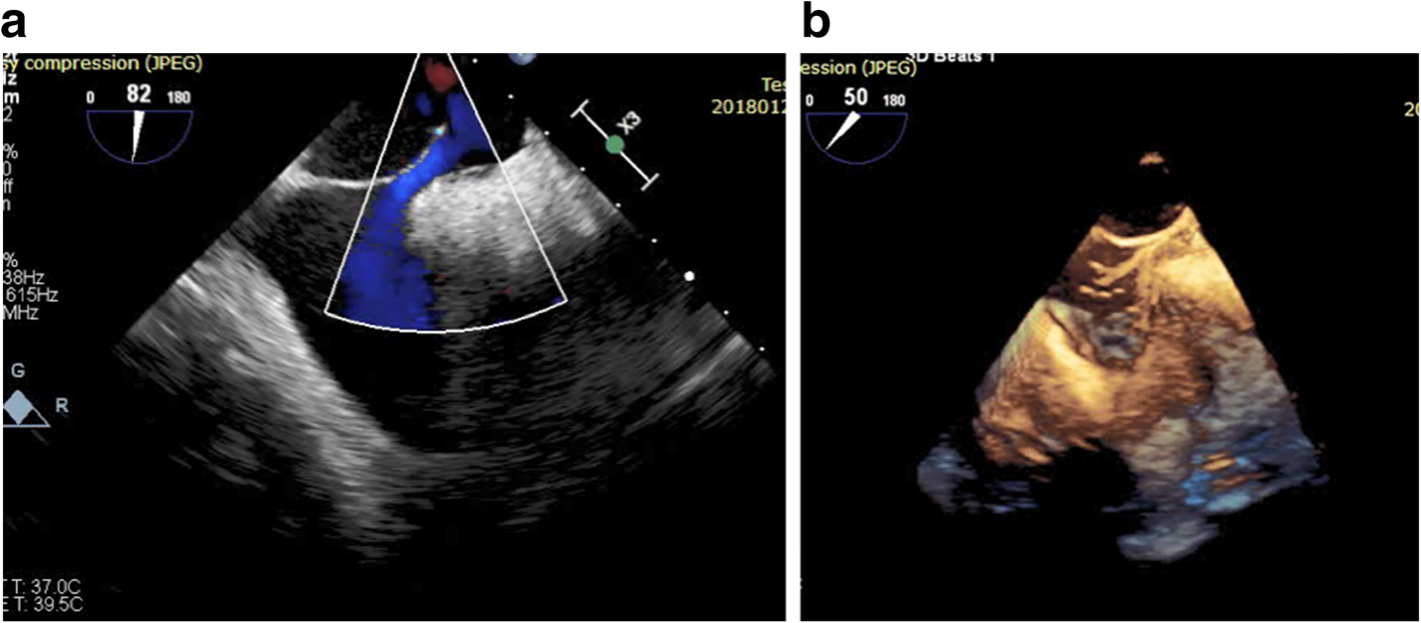
**a** 2D trans-esophageal echocardiography with color flow mode in bicaval view showing the wire across the patent foramen ovale (PFO). **b** Live 3D trans-esophageal echocardiography in bicaval view showing the wire across the patent foramen ovale (PFO)

**Fig. 4:**
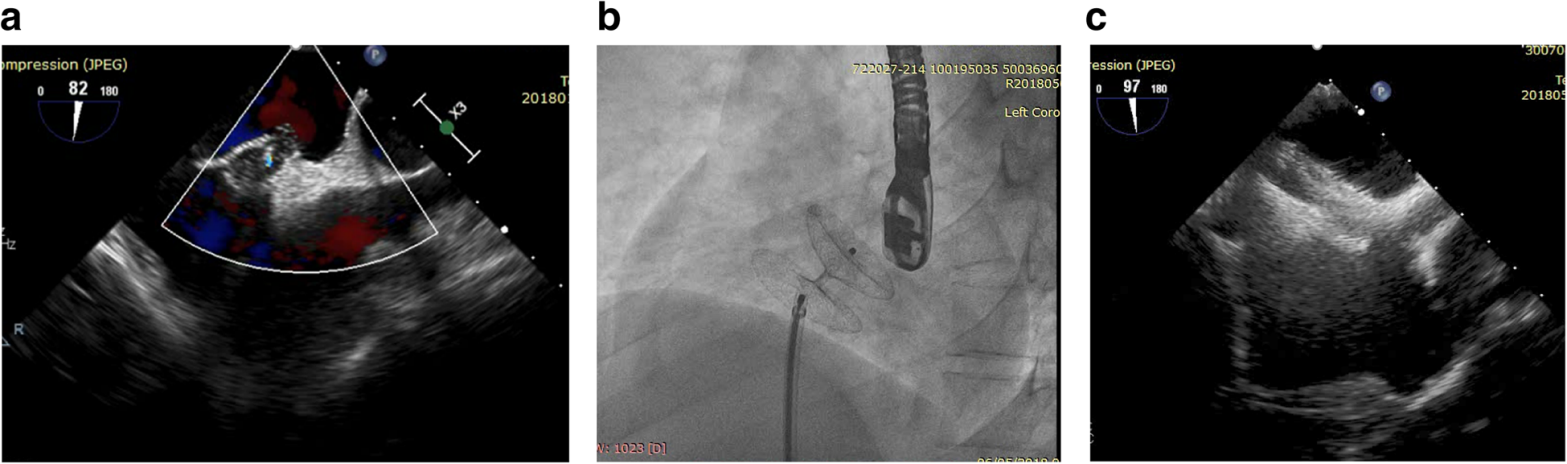
**a** 2D trans-esophageal echocardiography in bicaval view showing balloon sizing of the patent foramen ovale (PFO). **b** LAO view showing the cribriform device while deployment. **c** 2D trans-esophageal echocardiography in bicaval view showing cribriform device in place

Five patients had PFO device (one 18-mm device and four 25-mm devices); 5 patients had atrial septal occluder device (ASO)—this was done in patients with large sized and/or long tunneled PFO—(two 14-mm devices, one 15-mm device, one 16-mm device, and one 12-mm device); 8 patients had cribriform device (five 25 mm, two 30 mm, and one 35 mm). We failed to cross the PFO in only one patient (Figs. 2a, b, 3a, b, 4a, b, c).

### Incidence of complications

The procedure was successful in 18 patients (94.7%). None of the patients had major procedure-related complications; we did not experience any cases of device embolization or device erosion.

We encountered one case of atrial fibrillation that occurred during catheter manipulation within the left atrium while device deployment; it was successfully cardioverted using single synchronized DC shock. Cardiac monitoring of this patient at follow-up did not show any recurrent paroxysmal attacks.

**Fig. 5:**
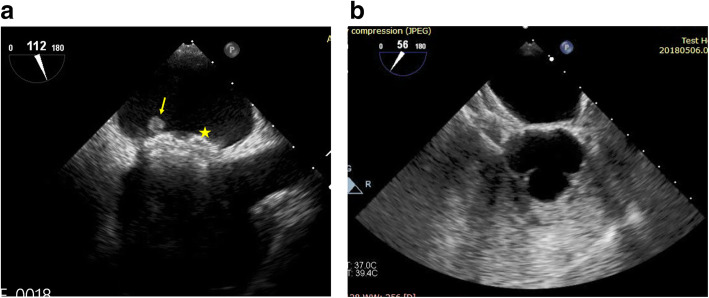
**a** 2D trans-esophageal echocardiography in bicaval view showing device in place (asterisk) and attached thrombus to the left atrial disc (arrow). **b** 2D trans-esophageal echocardiography in short axis mid-esophageal view showing device in place with bubbles filling the right side and no bubbles detected in the left side

We had one patient with device-related thrombosis on the left side disc (Fig. 5a). It was discovered 1 week after the procedure and was managed successfully by triple therapy (DAPT and oral anticoagulation) with no embolic events, and complete resolution of the thrombus at the follow-up as evidenced by trans-esophageal echocardiography study.

One patient had a TIA within the first 3 months after PFO device closure; this was managed by addition of anticoagulation to the patient’s medical treatment during the first 6 month after the procedure, with no recurrent events thereafter.

### Follow-up after device closure

Clinical follow-up every month for the first 6 months was done for all patients.

Transthoracic echocardiography was done the day next to the procedure, at 1 week, at 1 month, 3 months, and at 6 months.

TTE and/or TEE with bubble study was done to all patients at 6 months (Fig. 5b). c-TCD was done in patients with equivocal or positive bubble study results.

Absence of shunt was successfully achieved in 15 patients (83.3%) at 6 months as evidenced by bubble study and/or c-TCD.

Three patients showed significant reduction of the amount of shunt without complete abolishment. Those three patients had severe (grade III-curtain) right to left shunt at rest by c-TCD during their baseline assessment. They were all managed conservatively with extended DPAT without new clinical events.

All the patients were kept on DAPT therapy for 6 months, then single antiplatelet therapy for another 6 months. Patients were followed up regularly to estimate their bleeding and thrombotic risks; continuous evaluation by the stroke team members was done to determine the best anti-thrombotic therapy.

## Discussion

Stroke is a leading cause of death and disability worldwide with huge economic and psychosocial burden [1]. It is estimated that one third of transient ischemic attacks (TIAs) and ischemic strokes are of undetermined cause (i.e., cryptogenic). This leads to potential undermining of the secondary prevention [12].

Zahn first described paradoxical embolus in 1881, i.e., the translocation of a venous thrombus to the arterial circulation secondary to hemodynamic conditions which opens the PFO resulting in an embolic stroke. This mechanism is supported by several case studies showing thrombus across a PFO [3].

The earliest randomized trials of PFO closure like CLOSURE I and PC Trial did not demonstrate a superiority of closure compared with medical therapy. However, these findings were confounded by limited power, high rate of crossover between groups, failure to randomize the patients appropriately, and inconsistent use of anticoagulation in the medical arm of the trials. However, further randomized trials had overcome the previous limitations, and they have demonstrated the superiority of PFO closure over medical therapy in the prevention of recurrent stroke [3].

Early results of the RESPECT trial were neutral for PFO closure, but extended follow-up of patients demonstrated a significant reduction in the recurrence of ischemic stroke when compared to medical therapy *p* = 0.046, number needed to treat [NNT] 45. Furthermore, the REDUCE trial demonstrated also the superiority of PFO closure *p* = 0.002 when compared with antiplatelet therapy alone; the DEFENSE PFO study showed that PFO closure reduced significantly the composite endpoint of stroke, vascular death, and thrombolysis in myocardial infarction major bleeding at 2 years follow-up when compared with medical therapy. Finally, the CLOSE trial showed that none of the patients that received PFO closure experienced an ischemic stroke compared with 14 patients in the antiplatelet group [3].

Recent meta-analyses of these trials confirmed the superiority of PFO closure in adult patients with PFO-related stroke to reduce the risk of stroke recurrence [3].

These data urged the need for a collaborative cooperation between the neurologist and cardiologist for establishing a dedicated multidisciplinary cryptogenic stroke team to provide the best possible management to those patients in order to reduce the burden of the disease and risk of recurrence.

Active involvement of the patients in the decision of the management plan is mandatory to respect the patient’s preferences.

The incidence of patent foramen ovale was higher in patients with cryptogenic stroke, but in order to prove the active participation of the PFO in the pathogenesis of the stroke, cryptogenic stroke team needs scrutinized examination and thorough investigations to exclude other possible etiologies, and document the presence of right to left shunt.

Careful patient selection for transcatheter PFO closure is the key process; the procedure should only be done after a direct causation between the PFO and the TIA/stroke is documented to avoid exposing the patient to the procedural risk without any proven benefit.

This is the first formal, structured, dedicated program in Egypt, with the collaboration of multiple subspecialties to form a stroke team.

The results from our initial experience in transcatheter PFO closure in the context of patients with PFO-related stroke demonstrated the safety and effectiveness of the procedure. It also emphasized the need of a dedicated team.

## Conclusion

The formation of a dedicated stroke team had added significantly to the evaluation and management of patients with stroke. Our initial experience showed that the creation of such team is feasible and needs active participation and cooperation between the referring neurologist and cardiologists.

Increasing the awareness among both cardiologist and neurologist in Egypt is mandatory with expanding the concept of stroke team into a nationwide structured program urgently needed to reduce the disease burden and risk of recurrence and to implement the best evidence-based practice to the patients.

Our initial experience in transcatheter PFO closure in adult patients with PFO-related stroke showed that the procedure is safe, effective, with low incidence of procedural-related complications and high success rate.

### Recommendation

Raising the degree of awareness among the neurologist and cardiologist with the latest evidence is needed to offer the patients with the best management with the formation of a nationwide structured program for cryptogenic stoke.

Recruitment of more patients with longer follow up to establish an Egyptian database.

### Limitations

The number of patients is relatively small.

Only procedural outcomes and short-term follow-ups were reported.

## Data Availability

The datasets used and/or analyzed during the current study are available from the corresponding author on reasonable request

## Abbreviations

PFO: Patent foramen ovale
ASA: Atrial septal aneurysm
ASD: Atrial septal defect
TIA: Transient ischemic attack
c-TCD: Contrast transcranial Doppler
TTE: Transthoracic echocardiography
TEE: Trans-esophageal echocardiography
DAPT: Dual anti-platelet therapy
ANA: Anti-nuclear antibodies
Anti-DNA: Anti-deoxyribonucleic acid antibodies
ANCA: Antineutrophil cytoplasmic antibodies
IAS: Interatrial septum
SLE: Systemic lupus erythematosus
CBC: Complete blood count
ESR: Erythrocyte sedimentation rate
CRP: C-reactive protein
FBS: Fasting blood sugar
NIHSS: NIH stroke scale
mRs: Modified Rankin scale
ECG: Electrocardiogram
CTA: Computed tomography angiography
MRA: Magnetic resonance angiography
MRI: Magnetic resonance imaging brain
NNT: Number needed to treat
